# Lateral flow immunoassay for on-site detection of *Xanthomonas arboricola* pv. *pruni* in symptomatic field samples

**DOI:** 10.1371/journal.pone.0176201

**Published:** 2017-04-27

**Authors:** Pablo López-Soriano, Patricia Noguera, María Teresa Gorris, Rosa Puchades, Ángel Maquieira, Ester Marco-Noales, María M. López

**Affiliations:** 1 Centro de Protección Vegetal, Instituto Valenciano de Investigaciones Agrarias, Moncada, Valencia, Spain; 2 Instituto Universitario de Reconocimiento Molecular y Desarrollo Tecnológico, Departamento de Química, Universitat Politècnica de València, València, Spain; Oklahoma State University, UNITED STATES

## Abstract

*Xanthomonas arboricola* pv. *pruni* is a quarantine pathogen and the causal agent of the bacterial spot disease of stone fruits and almond, a major threat to *Prunus* species. Rapid and specific detection methods are essential to improve disease management, and therefore a prototype of a lateral flow immunoassay (LFIA) was designed for the detection of *X*. *arboricola* pv. *pruni* in symptomatic field samples. It was developed by producing polyclonal antibodies which were then combined with carbon nanoparticles and assembled on nitrocellulose strips. The specificity of the LFIA was tested against 87 *X*. *arboricola* pv. *pruni* strains from different countries worldwide, 47 strains of other *Xanthomonas* species and 14 strains representing other bacterial genera. All *X*. *arboricola* pv. *pruni* strains were detected and cross-reactions were observed only with four strains of *X*. *arboricola* pv. *corylina*, a hazelnut pathogen that does not share habitat with *X*. *arboricola* pv. *pruni*. The sensitivity of the LFIA was assessed with suspensions from pure cultures of three *X*. *arboricola* pv. *pruni* strains and with spiked leaf extracts prepared from four hosts inoculated with this pathogen (almond, apricot, Japanese plum and peach). The limit of detection observed with both pure cultures and spiked samples was 10^4^ CFU ml^-1^. To demonstrate the accuracy of the test, 205 samples naturally infected with *X*. *arboricola* pv. *pruni* and 113 samples collected from healthy plants of several different *Prunus* species were analyzed with the LFIA. Results were compared with those obtained by plate isolation and real time PCR and a high correlation was found among techniques. Therefore, we propose this LFIA as a screening tool that allows a rapid and reliable diagnosis of *X*. *arboricola* pv. *pruni* in symptomatic plants.

## Introduction

*Xanthomonas arboricola* pv. *pruni* is the causal agent of bacterial spot disease of stone fruits and almond. It affects a wide range of *Prunus* species including peach (*P*. *persica*), plum (*P*. *salicina*), almond (*P*. *amygdalus*, syn. *P*. *dulcis*), apricot (*P*. *armeniaca*), and cherry (*P*. *avium*), as well as ornamental plants such as *P*. *davidiana* and *P*. *laurocerasus* [1, 2]. It is considered one of the most important bacterial pathogens affecting this group of species, causing substantial losses in susceptible cultivars [3, 4]. In fact, it is listed as a quarantine organism in the EU phytosanitary legislation (EU Council Directive 2000/29/EC), and on the European and Mediterranean Plant Protection Organization (EPPO) list. *X*. *arboricola* pv. *pruni* was first described in North America in 1903 on Japanese plum [5], and it has later been reported in the main stone fruit producing areas worldwide. In Europe, the disease was first reported in 1920 in Italy, where it is now considered endemic [6], and in the last decade it has emerged in France, the Netherlands, Spain, Switzerland, and some countries of Eastern Europe [7]. In Spain, *X*. *arboricola* pv. *pruni* was first detected in 2002 on Japanese plum, and since then several outbreaks have occurred in different Spanish regions, in both commercial orchards and nurseries, on different *Prunus* species [8]. Suitable diagnostic protocols, as well as effective control measures, are required to prevent the introduction and dissemination of this quarantine pathogen [9].

The main symptoms of bacterial spot disease of *Prunus*, observed on leaves, include necrotic and angular spots that may drop out giving a ‘shot-hole’ appearance to the leaf. On fruits, sunken lesions are typical and gum flow may occur, especially after hail lessions or rainy days or periods of high humidity. Twig lesions have a dark, slightly depressed, and water-soaked appearance, and may progress to dieback [1, 10]. Symptoms caused by *X*. *arboricola* pv. *pruni* are relatively similar to those caused in stone fruits by other bacterial species such as *Pseudomonas syringae* pv. *mors*-*prunorum*, *P*. *syringae* pv. *persicae*, *P*. *syringae* pv. *syringae*, and *P*. *viridiflava*, and by some fungi, viruses or abiotic factors, thereby increasing the need for an effective diagnosis that allows rapid and accurate detection of the pathogen [7]. Currently, the only international regulation concerning the diagnosis of bacterial spot disease is an EPPO standard from 2006 [10], which is based on the isolation of the pathogen in agar media and subsequent identification through biochemical tests, protein profiling (SDS-PAGE), fatty acid profiling (FAME), immunofluorescence (IF), and pathogenicity tests. All these techniques are effective and reliable, but also laborious and time-consuming. Although molecular methods are not included in the EPPO standard protocol, they are very useful for the specific detection of *X*. *arboricola* pv. *pruni*. The first PCR protocol targeting *X*. *arboricola* pv. *pruni* was developed by Pagani [11] and recently several new protocols of PCR and Bio-PCR [12], duplex PCR [13], multiplex PCR [4], real time SYBR Green I assay and Bio-PCR [14], and real time TaqMan PCR [9] have been published. Compared to conventional methods, these molecular techniques are more sensitive and reproducible, although they also present some drawbacks, since expensive equipment and qualified personnel are required.

Lateral flow immunoassay (LFIA), widely employed in the diagnosis of plant pathogens, is considered as an efficient tool used for ‘point-of-care’ or ‘in-field’ pathogen detection [15]. An LFIA typically consists of a nitrocellulose membrane strip on which pathogen-specific antibodies are immobilized. These specific antibodies are bound to nanoparticles that are often made of colloidal gold, latex or silica to facilitate visual detection [16, 17].

LFIA has been used for different purposes, including diagnosis of human diseases, detection of toxic compounds in food, pregnancy tests, and in environmental settings [16]. Their use in phytopathology was focused mostly on fungi [18, 19] and viruses [20, 21], although it has been extended to the specific detection of bacteria such as *Erwinia amylovora* [22], *Clavibacter michiganensis* subsp. *sepedonicus* [23], and *Xanthomonas campestris* pv. *musacearum* [24]. LFIA offers several advantages over traditional techniques used in routine diagnostic procedures, such as its low cost, simplicity of use, and long shelf life; moreover, results are obtained within 10 minutes and tests can be performed on site by minimally trained staff [15, 17, 25]. It has become a reliable tool for the rapid screening of suspicious samples.

A wide range of nanoparticles can be used for LFIA and, among them, carbon nanoparticles have characteristics that make them a good alternative to other particles used in diagnostic applications [16]. They can be conjugated to a variety of biomolecules such as DNA, antibodies, and other proteins, thus allowing the detection of a variety of analytes. Moreover, they are economical and stable over time, and conjugates are easy to prepare (with no need for activation) and usually generate sensitive LFIA immunoassays [16]. Moreover, background noise is minimal (since there is little non-specific binding to the carbon particles, thus increasing the signal) and LFIA strips can be interpreted easily by visual inspection (a black line on a white background). For these reasons carbon nanoparticles were selected to manufacture the prototype of LFIA strips described in this work.

The aim of this study was to develop and validate a simple, rapid, and reliable lateral flow immunoassay for the detection of *X*. *arboricola* pv. *pruni* in field samples. A lateral flow device prototype was designed and polyclonal antibodies against *X*. *arboricola* pv. *pruni* were generated and conjugated with carbon nanoparticles. Specificity and sensitivity of the new device were determined, and finally it was compared with standard diagnostic methods in field samples.

## Materials and methods

### Bacterial strains and growth conditions

The bacterial strains used in this study are listed in S1 Table. To determine LFIA sensitivity, *X*. *arboricola* pv. *pruni* strains IVIA 2626.1, IVIA 3162.1, and IVIA 4430 were used for *in vitro* assays. The strain IVIA 3162.1 was selected for preparing spiked samples in leaf extracts. All bacteria were routinely grown on yeast peptone glucose agar (YPGA) medium [26] (yeast extract 5 g l^-1^ [Difco], bacteriological peptone 5 g l^-1^ [Difco], glucose 10 g l^-1^ and agar 20 g l^-1^ [pH 7.1]), and incubated at 25°C for 48 h.

### Production and characterization of the antisera against *X*. *arboricola* pv. *pruni*

#### Preparation of antigens

Bacterial antigens were generated with the *X*. *arboricola* pv. *pruni* Spanish strain IVIA 2626.1. Bacterial cultures were grown for 48 h on YPGA medium, and suspensions of 10^9^ CFU ml^-1^ in 10mM phosphate-buffered saline (PBS) were used to prepare antisera from two types of antigens, whole cells (WC) and heat-treated cells (HT). Heat-treated cells were obtained by incubating 1 ml aliquots of the suspensions at 100°C for 10 min.

#### Antiserum production and characterization

Two antisera (2626.1-WC and 2626.1-HT) were prepared. Female rabbits (Californian-New-Zealander cross) ca. 2 kg in weight were injected intramuscularly weekly for 4 weeks with 2 ml of a 1:1 emulsion of bacterial antigen with Freund’s incomplete adjuvant (Sigma-Aldrich, Madrid, Spain). The animals were bled 3 days after the final injection. The antisera were mixed with 50% of glycerol, sterilized by filtration, and stored at –80°C. Titers of 1:20000 and 1:80000 were determined by indirect-ELISA [27] for 2626.1-WC and 2626.1-HT, respectively.

#### Ethics statement

The rabbits used to produce the antibodies described here were housed and handled at the Instituto Valenciano de Investigaciones Agrarias (IVIA) in 2003 in accordance with the European legislation regarding animal welfare in research at that moment (Council Directive 86/609/EEC of 24 November 1986 on the approximation of laws, regulations, and administrative provisions of the Member States regarding the protection of animals used for experimental and other scientific purposes).

At the time when the experiments were performed, no animal Ethics Committee could review the protocol because such committees had not yet been created in our country. However, the protocols and experiments described in the paper were reviewed and approved by the former IVIA director and researchers with experience in the area at that time.

At the end of the experiment, animals were kept alive according to the requirements of the Council Directive 86/609/ECC for their use in subsequent experiments, and they received appropriate care under the supervision of a competent specialist.

### Lateral flow immunoassay

The reagents used to manufacture the carbon conjugate and LFIA strips were: H_3_BO_3_ and Tween 20 (Merck, Darmstadt, Germany); Na_2_B_4_O_7_⋅10 H_2_O, bovine serum albumin (BSA) (A2153), and polyclonal anti-rabbit antiserum IgG (R5506) (Sigma-Aldrich, Madrid, Spain). Antibodies were purified prior to their use with Hitrap GHP columns (GE Healthcare, Uppsala, Sweden) following the manufacturer’s instructions.

#### Carbon conjugate

Carbon nanoparticles were conjugated with polyclonal 2626.1-WC antibodies following a previously described methodology [25, 28]. Briefly, 10 mg of carbon nanoparticles (Spezial Schwartz 4, Degussa AG, Frankfurt, Germany) were suspended in 1 mL of MilliQ water and sonicated for 5 min (Branson model 250 Sonifier, Danbury, CT, USA). The resulting 1% (w/v) carbon suspension was diluted fivefold in borate buffer (BB) 5mM pH 8.8 and sonicated for 5 min. Next, 350 μg 2626.1-WC antibody was added to 1 mL of the diluted carbon suspension and the mixture was stirred for 3 h at room temperature. This suspension was first centrifuged at 14000 g for 15 min and the supernatant was removed. Then the pellet was washed with 1 ml of washing buffer (WB, 5 mM BB with 1% (w/v) BSA) to eliminate unbound protein. The mixture was centrifuged at 14000 g for 15 min at room temperature, the supernatant was removed, and the pellet was resuspended in 1 mL of WB. This process was repeated twice. After the final washing, the pellet was resuspended in 1 mL of 100 mM BB with 1% (w/v) BSA and stored at 4°C until use. The resulting homogeneous carbon nanoparticle suspension (CNP) contained 0.2% (w/v) carbon conjugate.

#### LFIA strips

A test line of 2626.1-HT antibody (0.2 mg/mL) was dispensed with a Biodot AD 1500 dispenser (Biodot, Irvine, California, USA) on a nitrocellulose (NC) membrane (HiFlow Plus HFB135, Millipore, Billerica, MA, USA). In order to validate the correct performance of lateral flow tests, a control line of an anti-rabbit antibody (0.5 mg/mL) was also dispensed onto the same NC strip, 5 mm from the first line of antibodies. Both immunoreagents were deposited on the NC membrane at a concentration of 1 μL/5 mm (diluted in 5 mM BB). After drying the NC membranes overnight at 37°C, they were fixed on plastic backing cards coated with a pressure-sensitive adhesive (G&L, Glen Rock, PA, USA) along with a cellulose absorbent pad (Schleicher and Schuell, ‘s-Hertogenbosch, the Netherlands). Finally, strips were cut to a length of 6 cm and a width of 5 mm using a Bio-Dot Cutter CM4000 (Irvine, CA, USA), and stored inside a desiccation chamber (in the dark) at room temperature until use.

#### LFIA assays

Wells of low-binding 96-well microplates (Nunc, MicroWell, Thermo Scientific, Madrid, Spain) were used to perform the LFIA assays. In each well, 1 μl of bacterial suspension was introduced along with 99 μl of a dilution of the carbon nanoparticle suspension (0,2% CNP diluted 1/100 with 100 mM BB containing 1% (w/v) BSA and 0.05% (v/v) Tween 20, and then sonicated for 15 seconds). After mixing the contents of the well, a strip was dipped in the well and left to stand vertically. This allowed the reagents to move upwards through the strip by capillary force and to react with the *X*. *arboricola* pv. *pruni* or rabbit antibodies. Strips were examined visually after 10 min; a positive result (+, presence) was recorded when the test line was distinguishable from the background by the naked eye, and a negative result (−, absence) was noted when no line was seen.

### Specificity assays

The specificity of the LFIA was determined using 87 *X*. *arboricola* pv. *pruni* strains, isolated in nine countries worldwide (Argentina, Australia, Brazil, Canada, France, Italy, New Zealand, Spain, and United States) and conserved in international collections. Strains were obtained from different hosts including almond (*P*. *amygdalus*, syn. *P*. *dulcis*), apricot (*P*. *armeniaca*), European plum (*P*. *domestica*), Japanese plum (*P*. *salicina*), nectarine (*P*. *persica* var. *nectarina*), peach (*P*. *persica*), and the hybrid *P*. *persica* x *P*. *dulcis*. In addition, 21 strains of *X*. *arboricola* of three other pathovars (*corylina*, *fragariae*, and *juglandis*), 26 strains of other *Xanthomonas* species (*X*. *alfalfae* pv. *citrumelonis*, *X*. *axonopodis* pv. *phaseoli*, *X*. *campestris* pv. *campestris*, *X*. *citri*, subsp. *citri* and *Xanthomonas* sp.), and 14 strains representing other species from different bacterial genera also pathogenic to stone fruit were included in the specificity assay. Bacterial cells from a 48 h culture in YPGA medium were resuspended in 10 mM phosphate buffered saline (PBS) at 10^8^ CFU ml^-1^. Tests with LFIA were performed as described above (LFIA assay section). Bacterial suspensions were heat treated at 95°C for 10 min and tested again. Assays were repeated twice.

### Sensitivity assays

Sensitivity was determined using serial 10-fold dilutions of PBS suspensions of three *X*. *arboricola* pv. *pruni* strains (IVIA 2626.1, IVIA 3162.1, and IVIA 4430) at a range of concentrations from 10 to 10^8^ CFU ml^-1^. Dilutions were heat treated at 95°C for 10 min and sensitivity was tested again. Additionally, sensitivity was evaluated in spiked samples, created by adding dilutions of the strain IVIA 3162.1 in leaf extracts of almond, apricot, Japanese plum, and peach. Extracts were prepared in accordance with Palacio-Bielsa *et al*. [9], i.e., by washing approximately 1 g fresh weight of mature, pathogen-free leaves with 15 ml of PBS in sterile plastic bags at room temperature for 15 min. Aliquots of 1 ml of each extract were inoculated with the strain at the same range of concentrations used for the pure cultures. Tests with LFIA were performed as described above, and the limit of detection was determined as the lowest concentration that produced a visible positive test line. Experiments were performed twice.

### Comparison of LFIA with plate isolation and real time PCR for detection of *X*. *arboricola* pv. *pruni* in naturally infected samples

Plant samples included in these analyses were collected in 2012 and 2013 from different stone fruits producing areas in Spain. The bacterial strains used in this study were obtained from samples received at the Laboratory of Reference (IVIA) of the Spanish Ministry of Agriculture. Symptomatic samples were processed in accordance with the EPPO protocol [10] with some modifications. On peach and apricot fruits, 1 cm^2^ pieces of tissue from the margin of the lesion were comminuted in small particles using sterile scalpels in sterile Petri dishes with 4.5 ml of PBS, and the suspension was left to stand for 1–2 min at room temperature. Almond fruits were washed in sterile plastic bags with 15 ml of PBS [29] for 5–15 min. Leaf samples were processed alternatively by washing approximately 1 g of fresh weight of leaves in 15 ml of PBS for 15 min, or cutting small pieces having 2–3 necrotic spots, in 4.5 ml of PBS in sterile Petri dishes and leaving them to stand for 5–15 min.

In addition, samples collected from healthy plants in zones having no history of *X*. *arboricola* pv. *pruni* were included in the analysis. Plant material consisted of leaves of almond, apricot, Japanese plum, peach, peach rootstock GF-305, *Prunus laurocerasus*, and the hybrid GF-677 (*P*. *dulcis* x *P*. *persica*). Healthy almond fruits were also included. All these samples were processed by washing as described above.

Two aliquots (1 ml each) were removed from each sample to use in parallel LFIA and bacterial cultivation. The remainder was stored at -20°C until it was used for real time PCR. Plate isolation was performed by streaking 50 μl of the extract on YPGA, and checking for the presence of suspected colonies after 48–72 h incubation at 25°C, which were then identified by PCR using the protocol described below.

Real time PCR assays were performed using Xap-2F (5’- TGG CTT CCT GAC TGT TTG CA- 3’) and Xap-2R (5’- TCG TGG GTT CGC TTG ATG A- 3’) primer set, in combination with the TaqMan probe Xap-2P (5’- 6-carboxyfluorescein [FAM]- TCA ATA TCT GTG CGT TGC TGT TCT CAC GA- 6-carboxytetramethylrhodamine [TAMRA]- 3’) [9], using a Light Cycler 480 (Roche, Manheim, Germany). The reaction mix contained: 2.5 μl sample, 0.4 μM each primer, 12.5 μl master mix (QuantiMix Easy Probes kit, Biotools, Madrid, Spain), and 150 nM TaqMan probe in a final volume of 25 μl. Real time PCR conditions were: an initial denaturation step at 95°C for 5 min, followed by 45 cycles, each one consisting of 1 min at 95°C and 1 min at 59°C. The expected product was a DNA fragment of 72 bp as indicated in Palacio-Bielsa *et al*. [9]. Appropriate negative controls including non-spiked leaf extracts of the four plant hosts, and master-mix-only samples were used in all reactions. Heat-treated suspensions of the strain IVIA 3162.1 at 10^7^ CFU ml^-1^ were included as positive controls.

In order to compare the results of the different diagnostic methods used in this study, and to evaluate the usefulness of the LFIA as a new diagnostic tool, contingency tables were calculated. Results obtained with the LFIA were compared independently with those obtained by plate isolation and real time PCR, both of which are considered ‘gold standard’ methods [22], and parameters such as diagnostic specificity, diagnostic sensitivity, false positive and negative ratings, and relative accuracy were calculated in accordance with Olmos *et al*. [30] and the EPPO standards PM 7/98(2) [31].

## Results

### Specificity assays

To assess the specificity of the LFIA, a wide range of *Xanthomonas* species and other bacterial species were tested. The 87 *X*. *arboricola* pv. *pruni* strains, representing isolates from nine countries and seven hosts, were detected. Cross-reactions were observed only with four strains of *X*. *arboricola* pv. *corylina* (CFBP 1846, RIPF-X10, RIPF-X18, and RIPF-X23), a hazelnut pathogen not reported in stone fruit or almond trees. All the other *Xanthomonas* strains tested were negative, as were the rest of the bacterial species included in the assay (Table 1). Exactly the same results were obtained with the heat-treated bacterial suspensions.

**Table 1:** Specificity of the lateral flow immunoassay (LFIA) against strains of *Xanthomonas arboricola* pv. *pruni* (n = 87), other *Xanthomonas* species (n = 47), and other bacterial species related with stone fruits (n = 14).

Species	Host	LFIA (Positive samples/Total samples)
*Xanthomonas arboricola* pv. *pruni*	*Prunus* spp.	87/87
*X*. *arboricola* pv. *corylina*	*Corylus avellana*	4/6
*X*. *arboricola* pv. *fragariae*	*Fragaria* spp.	0/2
*X*. *arboricola* pv. *juglandis*	*Juglans regia*	0/11
*X*. *arboricola*	*P*. *persica*	0/2
*X*. *alfalfae* pv. *citrumelonis*	*C*. *paradisi x Poncirus trifoliata*	0/1
*X*. *axonopodis* pv. *phaseoli*	*Phaseolus vulgaris*	0/1
*X*. *campestris* pv. *campestris*	*Brassica oleracea*	0/2
*X*. *citri* subsp. *citri*	*Citrus sinensis*	0/12
*Xanthomonas* sp.	*Capsicum annuum/Prunus* spp.	0/10
*Agrobacterium tumefaciens*	*Prunus* spp.	0/5
*Pantoea agglomerans*	*Olea europea*	0/1
*Pseudomonas fluorescens*	Several	0/3
*P*. *syringae*	Several	0/4
*P*. *syringae* pv. *actinidae*	*Actinidia deliciosa*	0/1

### Sensitivity assays

**Fig 1:**
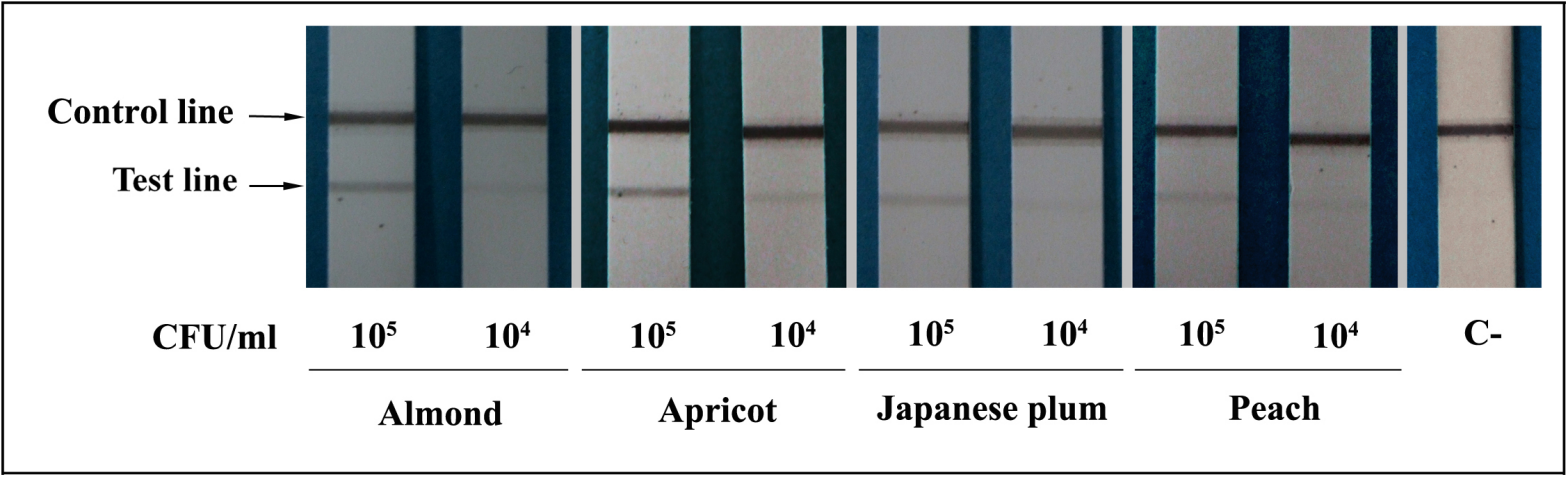
Sensitivity of the lateral flow immunoassay in spiked samples. Plant extracts of four *Xanthomonas arboricola* pv. *pruni* hosts were inoculated with serially dilutedf suspensions of the strain IVIA 3162.1. Bacterial concentrations are indicated as CFU ml^-1^. A non-spiked negative control is indicated as C-.

The sensitivity of the LFIA was first tested using serial dilutions from pure cultures of several *X*. *arboricola* pv. *pruni* strains (IVIA 2626.1, IVIA 3162.1, and IVIA 4430) obtaining a limit of detection of 10^4^ CFU ml^-1^. On comparing heat-treated and untreated suspensions no differences were observed. In addition, sensitivity was also assessed in spiked samples, in which serially diluted suspensions of the strain IVIA 3162.1 were added to leaf extracts of almond, apricot, Japanese plum, and peach. Sensitivity, expressed as the lowest amount of pathogen detected, was the same in the four hosts. In all of them, the limit of detection was 10^4^ CFU ml^-1^ (Fig 1), the same as that obtained with pure cultures. Nevertheless, suspensions having titers lower than 10^5^ CFU ml^-1^ showed weak positive test lines, which became more obvious after the membranes were dried.

### Comparison of LFIA with plate isolation and real time PCR for detection of *X*. *arboricola* pv. *pruni* in naturally infected plant samples

A total of 205 symptomatic samples collected from three *Prunus* species (almond, peach, and apricot) showing typical *X*. *arboricola* pv. *pruni* symptoms were analyzed in the laboratory with both ‘gold standard’ (plate isolation and real time PCR methods) and also tested with the LFIA (Table 2). Almond and peach samples included both symptomatic fruits and leaves, whereas apricot samples consisted only of fruits. All these symptomatic samples gave a positive identification by plate isolation and real time PCR, whereas eight were negative by LFIA (five almond leaves, one peach leaf, and two apricot fruits).

**Table 2:** Comparison of lateral flow immunoassay (LFIA), plate isolation and real time PCR for detection of *Xanthomonas arboricola* pv. *pruni* in naturally infected plant samples.

		Positive samples / Total samples		
Host	Organ	Plate isolation	Real time PCR	LFIA
Almond	• Fruit • Leaf	• 31/31 • 38/38	• 131/131 • 38/38	• 131/131 • 33/38
Peach	• Fruit • Leaf	• 20/20 • 10/10	• 20/20 • 10/10	• 20/20 • 9/10
Apricot	Fruit	6/6	6/6	4/6
**Total**		**105/105**	**205/205**	**197/205**

Additionally, in order to calculate the diagnostic parameters of the LFIA, 113 samples collected from healthy plants of seven *Prunus* species or hybrids were also processed with the three methods. All the samples from healthy plants were negative with the three methodologies (Table 3).

**Table 3:** Comparison of lateral flow immunoassay (LFIA), plate isolation and real time PCR for detection of *Xanthomonas arboricola* pv. *pruni* in asymptomatic samples collected from healthy plants.

		Positive samples / Total samples		
Host	Organ	Plate isolation	Real time PCR	LFIA
Almond	• Fruit • Leaf	• 0/10 • 0/20	• 0/10 • 0/20	• 0/10 • 0/20
Peach	Leaf	0/15	0/15	0/15
Apricot	Leaf	0/15	0/15	0/15
Japanese plum	Leaf	0/18	0/18	0/18
GF-305	Leaf	0/10	0/10	0/10
GF-677	Leaf	0/15	0/15	0/15
*Prunus laurocerasus*	Leaf	0/10	0/10	0/10
**Total**		**0/113**	**0/113**	**0/113**

Results obtained with LFIA were compared with those obtained with real time PCR (Table 4). Out of 318 samples processed, 197 were positive by LFIA, whereas 205 were positive using the real time PCR. All the samples collected from healthy plants were negative by both techniques. Thus, a diagnostic specificity of 100% and diagnostic sensitivity of 96.1% were obtained when comparing both techniques, with a low rate of false negatives (3.9%). False positives were not detected in our samples. The relative accuracy obtained between techniques was 97.5%. Other diagnostic parameters calculated are shown in Table 4.

**Table 4:** Contingency table comparing lateral flow immunoassay (LFIA) with real time PCR for *Xanthomonas arboricola* pv. *pruni* detection in samples of naturally infected and healthy plants. The upper part shows the positive and negative results for each technique. The diagnostic parameters corresponding to these results are shown in the lower part.

	Real time PCR			
**Lateral flow immunoassay**		Positive	Negative	Total
	Positive	197	0	197
	Negative	8	113	121
	Total	205	113	318
**Diagnostic parameters**^a^	Diagnostic sensitivity			96.1%
	Diagnostic specificity			100%
	Positive predictive value			100%
	Negative predictive value			93.4%
	False positive rate			-
	False negative rate			3.9%
	Prevalence rate			64.5%
	Likelihood ratio for positive results			-
	Likelihood ratio for negative results			0.04
	Relative accuracy			97.5

^a^ Diagnostic parameters calculated in accordance with Olmos *et al*. [30] and the EPPO standards PM 7/98(2) [31] are shaded in orange

The LFIA was also compared to plate isolation (Table 5). Out of 218 samples included, 97 were positive using the LFIA and 105 were positive by plate isolation. All samples obtained from healthy plants were also negative by plate isolation. The diagnostic sensitivity of the LFIA compared to isolation was slightly lower (92.4%) than the one previously obtained when comparing the LFIA with the real time PCR (96.1%), whereas diagnostic specificity was also 100%. The false negative rate obtained was 7.6% and no false positives were detected. Relative accuracy between LFIA and plate isolation was 96.3%. As before, other parameters calculated are shown in Table 5.

**Table 5:** Contingency table comparing lateral flow immunoassay (LFIA) with plate isolation for *Xanthomonas arboricola* pv. *pruni* detection in samples of naturally infected and healthy plants. The upper part shows the positive and negative results for each technique. The diagnostic parameters corresponding to these results are shown in the lower part.

	Plate isolation			
Lateral flow immunoassay		Positive	Negative	Total
	Positive	97	0	97
	Negative	8	113	121
	Total	105	113	218
**Diagnostic parameters**^a^	Diagnostic sensitivity^a^			92.4%
	Diagnostic specificity			100%
	Positive predictive value			100%
	Negative predictive value			93.4%
	False positive rate			-
	False negative rate			7.6%
	Prevalence rate			48.2%
	Likelihood ratio for positive results			-
	Likelihood ratio for negative results			0.08
	Relative accuracy			96.3

^a^ Diagnostic parameters calculated in accordance with Olmos *et al*. [30] and the EPPO standards PM 7/98(2) [31] are shaded in orange.

## Discussion

Bacterial spot disease is considered one of the main bacterial diseases affecting *Prunus* species and effective management strategies may cause a considerable decrease in disease incidence. Development of rapid diagnostic methods for reliable detection of *X*. *arboricola* pv. *pruni*, the causal agent of this major disease, is essential to prevent its dissemination and establishment into new areas. The LFIA developed in this study provides an alternative diagnostic test that combines the advantages of enough specificity and sensitivity, low cost, and rapid and simple operation, which make it especially appropriate for on-site analysis of symptomatic *Prunus* field samples.

The LFIA was shown to be specific to *X*. *arboricola* pv. *pruni*, detecting all strains representing a worldwide collection. Cross-reactivity was observed only with four strains of *X*. *arboricola* pv. *corylina*, which is the causal agent of the bacterial blight of hazelnut [32]. This pathogen is phylogenetically highly related to *X*. *arboricola* pv. *pruni* [33] and in fact, primers designed for *X*. *arboricola* pv. *pruni* have been used for the identification of the pathovar *corylina*, since strains of this pathovar also amplify with them [13]. Interestingly, as *X*. *arboricola* pv. *corylina* has never been reported in *Prunus* species, the antibodies generated against *X*. *arboricola* pv. *pruni* can still be used for the detection of the bacterial spot pathogen in *Prunus* spp. None of the other bacteria tested were detected, including the rest of the *X*. *arboricola* strains and other bacterial species commonly isolated in stone fruits, thereby confirming the specificity of LFIA for the purpose of this work.

The detection limit obtained with the lateral flow immunoassay was 10^4^ CFU ml^-1^ both in pure cultures and spiked samples, a level that is sufficient to detect *X*. *arboricola* pv. *pruni* in symptomatic samples, in which bacterial populations are typically higher than 10^6^ CFU ml^-1^ [9, 34, 35]. This limit is similar to that reported with other lateral flow devices designed for the detection of bacterial pathogens, such as *X*. *campestris* pv. *musacearum* [24] or *C*. *michiganensis* subsp. *sepedonicus* [23], but better than the one obtained for *E*. *amylovora* [22]. In addition, in our hands the use of an available commercial kit (Pocket Diagnostic, Sand Hutton, York, UK) for on-site detection of *X*. *arboricola* pv. *pruni* revealed a limit of detection of 10^5^ CFU ml^-1^, which is lower than the one obtained with the prototype described in this study. Although 10^4^ CFU ml^-1^ can be considered a low sensitivity, it is enough for detection in symptomatic samples, which is the objective of our development.

The similarity between the detection limit obtained with pure cultures and that obtained with spiked samples suggests that there is no significant influence of the plant material in the sensitivity of the immunoassay. Leaf extracts of different *Prunus* species (almond, apricot, Japanese plum, and peach) were tested and no significant differences were found among them, demonstrating a general usefulness of LFIA for *X*. *arboricola* pv. *pruni* detection in all hosts. Unlike the limit of detection obtained with LFIA, other methods such as conventional PCR and real time PCR have lower sensitivity when used with Japanese plum samples compared to those of other hosts [7, 9], probably due to the presence of inhibitors in Japanese plum extracts. This may indicate that LFIA is not affected by the presence of potential inhibitors, like phenolic compounds, in leaf extracts, which is an advantage over other *X*. *arboricola* pv. *pruni* detection methodologies.

Heat-treated suspensions were also detected at the same sensitivity as with living bacteria, suggesting heat stability of the epitopes which can be detected by the antibodies obtained in this study. The use of this type of suspension facilitates the handling of this quarantine pathogen in some countries.

The LFIA was compared with two standard methodologies typically used for the detection and identification of *X*. *arboricola* pv. *pruni*, such as plate isolation and real time PCR. Diagnostic specificity of the LFIA was the same (100%) when compared to the other two techniques, whereas diagnostic sensitivity of the LFIA compared to plate isolation (92.4%) was slightly lower than the one obtained when compared to real time PCR (96.1%). However, the comparison of LFIA with plate isolation was based on only 105 naturally infected samples, due to the fact that isolation could not be performed in 100 almond fruits. This difference in the total number of samples analyzed by plate isolation compared to the total number of samples tested by real time PCR and LFIA could explain the lower diagnostic sensitivity of the LFIA with respect to plate isolation. Furthermore, having fewer samples analyzed by plate isolation probably contributed to the false negative rate obtained (7.6%) in contrast to that obtained when comparing LFIA to real time PCR (3.9%).

Although our results showed that all the samples were positive after plate isolation, it should be noted that colony appearance and/or counting may be hampered by the overgrowth of colonies of saprophytic bacteria commonly isolated from field samples. Additionally, plate isolation only detects culturable bacteria, whereas LFIA and real time PCR both detect dead and putative viable but non culturable (VBNC) bacteria. This state has been described in different *Xanthomonas* species such as *X*. *campestris* pv. *campestris* [36] and *X*. *citri* subsp. *citri* [37, 38]. *X*. *arboricola* pv. *pruni* cells are also very likely to enter into the VBNC state, potentially leading to an advantage of the LFIA over plate isolation.

Naturally infected samples used in this study included different plant species and different plant tissues, i.e., leaves (almond and peach) and fruits (peach, apricot, and almond). Samples that gave negative results by LFIA (n = 8) belonged to the different plant species analyzed (five from almond, two from apricot, and one from peach), suggesting the absence of any correlation between hosts and negative results.

Additionally, 113 samples collected from healthy plants, also processed by the three methodologies, all tested negative, thus confirming the absence of false positives.

A comparison of the relative accuracy obtained between LFIA and real time PCR (97.5%) and LFIA and plate isolation (96.3%) demonstrates a strong correlation between techniques. Moreover, the lateral flow immunoassay allows minimally trained users to obtain reliable results in less than 15 minutes and, unlike plate isolation and real time PCR, it can be used directly in the field as an initial screening tool to rule out other bacterial or fungal diseases causing similar symptoms.

## Supporting information

S1 Table Bacterial strains used in this study, their country of origin, host and results obtained with LFIA. (DOCX)
