# Supplementary material for: Lateral flow immunoassay for on-site detection of Xanthomonas arboricola pv. pruni in symptomatic field samples
Source: PLoS One. 2017 Apr 27;12(4):e0176201. doi: 10.1371/journal.pone.0176201 (PMC5407831; doi:10.1371/journal.pone.0176201)
Supplement: S1 Table — (DOCX) [file pone.0176201.s001.docx]

Table S1. Bacterial strains used in this study, their country of origin, host and results obtained with LFIA.

| **Strain^ab^** | **Species** | **Country of origin** | **Host species** | **LFIA^c^** |
| --- | --- | --- | --- | --- |
| IVIA 2626.1 | *Xanthomonas arboricola* pv. *pruni* | Spain | *Prunus salicina* | + |
| IVIA 2626.3 | *X. arboricola* pv. *pruni* | Spain | *Prunus salicina* | + |
| IVIA 2647.1-2 | *X. arboricola* pv. *pruni* | Spain | *Prunus salicina* | + |
| IVIA 2647.3-1 | *X. arboricola* pv. *pruni* | Spain | *Prunus salicina* | + |
| IVIA 2647.1-8b | *X. arboricola* pv. *pruni* | Spain | *Prunus salicina* | + |
| IVIA 2647.3-1b | *X. arboricola* pv. *pruni* | Spain | *Prunus salicina* | + |
| IVIA 2649.1 | *X. arboricola* pv. *pruni* | Spain | *Prunus salicina* | + |
| IVIA 2667 | *X. arboricola* pv. *pruni* | Spain | *Prunus salicina* | + |
| IVIA 2795 | *X. arboricola* pv. *pruni* | Spain | *Prunus salicina* | + |
| IVIA 2758.1 | *X. arboricola* pv. *pruni* | Spain | *Prunus salicina* | + |
| IVIA 2758.2 | *X. arboricola* pv. *pruni* | Spain | *Prunus salicina* | + |
| IVIA 2758.3 | *X. arboricola* pv. *pruni* | Spain | *Prunus salicina* | + |
| IVIA 2826.1 | *X. arboricola* pv. *pruni* | Spain | *Prunus persica* | + |
| IVIA 2826.6 | *X. arboricola* pv. *pruni* | Spain | *Prunus persica* | + |
| IVIA 2826.10 | *X. arboricola* pv. *pruni* | Spain | *Prunus persica* | + |
| IVIA 2832.4b | *X. arboricola* pv. *pruni* | Spain | *Prunus persica* | + |
| IVIA 2832.21 | *X. arboricola* pv. *pruni* | Spain | *Prunus persica* | + |
| IVIA 2832.27 | *X. arboricola* pv. *pruni* | Spain | *Prunus persica* | + |
| IVIA 2835.7 | *X. arboricola* pv. *pruni* | Spain | *Prunus salicina* | + |
| IVIA 2835.8 | *X. arboricola* pv. *pruni* | Spain | *Prunus salicina* | + |
| IVIA 2835.9 | *X. arboricola* pv. *pruni* | Spain | *Prunus salicina* | + |
| IVIA 3161.2 | *X. arboricola* pv. *pruni* | Spain | *Prunus dulcis* | + |
| IVIA 3162.1 | *X. arboricola* pv. *pruni* | Spain | *Prunus dulcis* | + |
| IVIA 3162.3 | *X. arboricola* pv. *pruni* | Spain | *Prunus dulcis* | + |
| IVIA 3162.4 | *X. arboricola* pv. *pruni* | Spain | *Prunus dulcis* | + |
| IVIA 3177.1-1 | *X. arboricola* pv. *pruni* | Spain | *Prunus dulcis* | + |
| IVIA 3181.3-1-6 | *X. arboricola* pv. *pruni* | Spain | *Prunus dulcis* | + |
| IVIA 3181.3-1-7 | *X. arboricola* pv. *pruni* | Spain | *Prunus dulcis* | + |
| IVIA 3181.3-3 | *X. arboricola* pv. *pruni* | Spain | *Prunus dulcis* | + |
| IVIA 3355.1 | *X. arboricola* pv. *pruni* | Spain | *Prunus salicina* | + |
| IVIA 3373.1a | *X. arboricola* pv. *pruni* | Spain | *Prunus salicina* | + |
| IVIA 3374.a | *X. arboricola* pv. *pruni* | Spain | *Prunus salicina* | + |
| IVIA 3378.2 | *X. arboricola* pv. *pruni* | Spain | *Prunus persica* var*. nectarina* | + |
| IVIA 3397.1 | *X. arboricola* pv. *pruni* | Spain | *Prunus salicina* | + |
| IVIA 3397.2 | *X. arboricola* pv. *pruni* | Spain | *Prunus salicina* | + |
| IVIA 3437.5 | *X. arboricola* pv. *pruni* | Spain | *Prunus salicina* | + |
| IVIA 3438.8 | *X. arboricola* pv. *pruni* | Spain | *Prunus salicina* | + |
| IVIA 3439.1 | *X. arboricola* pv. *pruni* | Spain | *Prunus salicina* | + |
| IVIA 3487.1 | *X. arboricola* pv. *pruni* | Spain | *Prunus armeniaca* | + |
| IVIA 3487.4 | *X. arboricola* pv. *pruni* | Spain | *Prunus armeniaca* | + |
| IVIA 3489.4 | *X. arboricola* pv. *pruni* | Spain | *Prunus salicina* | + |
| IVIA 3490.1 | *X. arboricola* pv. *pruni* | Spain | *Prunus persica* | + |
| IVIA 3490.2 | *X. arboricola* pv. *pruni* | Spain | *Prunus persica* | + |
| IVIA 3490.3 | *X. arboricola* pv. *pruni* | Spain | *Prunus persica* | + |
| IVIA 3490.4 | *X. arboricola* pv. *pruni* | Spain | *Prunus persica* | + |
| IVIA 3491.7 | *X. arboricola* pv. *pruni* | Spain | *Prunus persica* | + |
| IVIA 3491.8 | *X. arboricola* pv. *pruni* | Spain | *Prunus persica* | + |
| IVIA 3492.2 | *X. arboricola* pv. *pruni* | Spain | *Prunus persica* | + |
| IVIA 3492.3 | *X. arboricola* pv. *pruni* | Spain | *Prunus persica* | + |
| IVIA 3604.2 | *X. arboricola* pv. *pruni* | Spain | *Prunus dulcis* | + |
| IVIA 3704.2 | *X. arboricola* pv. *pruni* | Spain | *Prunus salicina* | + |
| IVIA 3704.6 | *X. arboricola* pv. *pruni* | Spain | *Prunus salicina* | + |
| IVIA 3704.7 | *X. arboricola* pv. *pruni* | Spain | *Prunus salicina* | + |
| IVIA 3705.6 | *X. arboricola* pv. *pruni* | Spain | *Prunus salicina* | + |
| IVIA 3705.7 | *X. arboricola* pv. *pruni* | Spain | *Prunus salicina* | + |
| IVIA 3741 | *X. arboricola* pv. *pruni* | Spain | *Prunus salicina* | + |
| IVIA 3742 | *X. arboricola* pv. *pruni* | Spain | *Prunus salicina* | + |
| IVIA 3767.1 | *X. arboricola* pv. *pruni* | Spain | *Prunus persica* | + |
| IVIA 3767.2 | *X. arboricola* pv. *pruni* | Spain | *Prunus persica* | + |
| IVIA 3767.3 | *X. arboricola* pv. *pruni* | Spain | *Prunus persica* | + |
| IVIA 4113 | *X. arboricola* pv. *pruni* | Spain | *Prunus salicina* | + |
| IVIA 4165.15 | *X. arboricola* pv. *pruni* | Spain | *Prunus dulcis* | + |
| IVIA 4166.12 | *X. arboricola* pv. *pruni* | Spain | *Prunus dulcis* | + |
| CFBP 411 | *X. arboricola pv. pruni* | USA | Unknown | + |
| CFBP 1311 | *X. arboricola* pv. *pruni* | Canada | Unknown | + |
| CFBP 3894^PT^ | *X. arboricola* pv. *pruni* | New Zealand | *Prunus salicina* | + |
| CFBP 5229 | *X. arboricola* pv. *pruni* | Argentina | *Prunus* sp. | + |
| CFBP 5529 | *X. arboricola* pv. *pruni* | Australia | *Prunus persica* | + |
| CFBP 5530 | *X. arboricola* pv. *pruni* | Italy | *Prunus persica* | + |
| CFBP 5562 | *X. arboricola* pv. *pruni* | France | *Prunus persica* | + |
| CFBP 5720 | *X. arboricola* pv. *pruni* | USA | *Prunus persica* | + |
| CFBP 5722 | *X. arboricola* pv. *pruni* | Brazil | *Prunus persica* | + |
| CFBP 5724 | *X. arboricola* pv. *pruni* | USA | *Prunus dulcis* | + |
| ISF 43 | *X. arboricola* pv. *pruni* | Italy | *Prunus salicina* | + |
| ISF 463 | *X. arboricola* pv. *pruni* | Italy | *Prunus persica* | + |
| ISF 464 | *X. arboricola* pv. *pruni* | Italy | *Prunus persica* | + |
| ISF 465 | *X. arboricola* pv. *pruni* | Italy | *Prunus persica* | + |
| ISF 515 | *X. arboricola* pv. *pruni* | Italy | *Prunus persica* | + |
| DAR 33337 | *X. arboricola* pv. *pruni* | Australia | *Prunus salicina* | + |
| DAR 33420 | *X. arboricola* pv. *pruni* | Australia | *Prunus persica* | + |
| DAR 41285 | *X. arboricola* pv. *pruni* | Australia | *Prunus persica* | + |
| DAR 41286 | *X. arboricola* pv. *pruni* | Australia | *Prunus persica* | + |
| DAR 41287 | *X. arboricola* pv. *pruni* | Australia | *Prunus persica* | + |
| DAR 56679 | *X. arboricola* pv. *pruni* | Australia | *Prunus armeniaca* | + |
| DAR 56680 | *X. arboricola* pv. *pruni* | Australia | *Prunus salicina* | + |
| DAR 61729 | *X. arboricola* pv. *pruni* | Australia | *Prunus domestica* | + |
| DAR 69849 | *X. arboricola* pv. *pruni* | Australia | *P. persica* x *P. dulcis* | + |
| CFBP 1846 | *X. arboricola* pv. *corylina* | France | *Corylus avellana* | + |
| IVIA 3978 | *X. arboricola* pv. *corylina* | Spain | *Corylus avellana* | - |
| RIPF-X08 | *X. arboricola* pv. *corylina* | Poland | *Corylus avellana* | - |
| RIPF-X10 | *X. arboricola* pv. *corylina* | Poland | *Corylus avellana* | + |
| RIPF-X18 | *X. arboricola* pv. *corylina* | Poland | *Corylus avellana* | + |
| RIPF-X23 | *X. arboricola* pv. *corylina* | Poland | *Corylus avellana* | + |
| CFBP 6771^PT^ | *X. arboricola* pv. *fragariae* | Italy | *Fragaria* sp. | - |
| IVIA 2063.2 | *X. arboricola pv. fragariae* | Spain | *Fragaria* sp. | - |
| IVIA 1317.1a | *X. arboricola* pv*. juglandis* | Spain | *Juglans regia* | - |
| IVIA 1321.1a | *X. arboricola* pv*. juglandis* | Spain | *Juglans regia* | - |
| IVIA 1325.3a | *X. arboricola* pv*. juglandis* | Spain | *Juglans regia* | - |
| IVIA 1327.4a | *X. arboricola* pv*. juglandis* | Spain | *Juglans regia* | - |
| IVIA 3114.1 | *X. arboricola* pv*. juglandis* | Spain | *Juglans regia* | - |
| IVIA 3114.2 | *X. arboricola* pv*. juglandis* | Spain | *Juglans regia* | - |
| IVIA 4254.1-1 | *X. arboricola* pv*. juglandis* | Spain | *Juglans regia* | - |
| IVIA 4254.8-4 | *X. arboricola* pv*. juglandis* | Spain | *Juglans regia* | - |
| RIPF-X04 | *X. arboricola* pv. *juglandis* | Poland | *Juglans regia* | - |
| RIPF-X05 | *X. arboricola* pv. *juglandis* | Poland | *Juglans regia* | - |
| RIPF-X06 | *X. arboricola* pv. *juglandis* | Poland | *Juglans regia* | - |
| IVIA 2835.1 | *X. arboricola* | Spain | *Prunus persica* | - |
| IVIA 4185 | *X. arboricola* | Spain | *Prunus persica* | - |
| F3 | *X. alfalfae* pv*. citrumelo* | USA | *Citrus paradisi x Poncirus trifoliata* | - |
| CECT-914 | *X. axonopodis* pv*. phaseoli* | Hungary | *Phaseolus vulgaris* | - |
| IVIA 2734.1 | *X. campestris* pv*. campestris* | Spain | *Brassica oleracea var. capitata* | - |
| IVIA 2808.7a | *X. campestris* pv*. campestris* | Spain | *Brassica oleracea var. botrytis* | - |
| IVIA 3222.6 | *X. citri* subsp. *citri* | Uruguay | *Citrus sinensis* | - |
| IVIA 2776.107 | *X. citri* subsp. *citri* | Argentina | *Citrus sinensis* | - |
| IVIA 2800.1Z | *X. citri* subsp. *citri* | Argentina | *Citrus sinensis* | - |
| IVIA 2808.8-1 | *X. citri* subsp. *citri* | Argentina | *Citrus sinensis* | - |
| IVIA 2807.10-2 | *X. citri* subsp. *citri* | Argentina | *Citrus sinensis* | - |
| IVIA 2889.1 | *X. citri* subsp. *citri* | Argentina | *Citrus sinensis* | - |
| IVIA 3011.3a | *X. citri* subsp. *citri* | Uruguay | *Citrus sinensis* | - |
| IVIA 3023.2 | *X. citri* subsp. *citri* | Uruguay | *Citrus sinensis* | - |
| IVIA 3024.5 | *X. citri* subsp. *citri* | Uruguay | *Citrus sinensis* | - |
| IVIA 3026.1 | *X. citri* subsp. *citri* | Uruguay | *Citrus sinensis* | - |
| AW | *X. citri* subsp. *citri* | USA | Unknown | - |
| IVIA 3365.13 | *X. citri* subsp. *citri* | Uruguay | *Citrus sinensis* | - |
| IVIA 3080 | *Xanthomonas* sp. | Spain | *Capsicum annuum* | - |
| IVIA 3081 | *Xanthomonas* sp. | Spain | *Capsicum annuum* | - |
| IVIA 3082 | *Xanthomonas* sp. | Spain | *Capsicum annuum* | - |
| IVIA 2636.1-1 | *Xanthomonas* sp. | Spain | *Capsicum annuum* | - |
| CECT-792 | *Xanthomonas* sp. | Israel | Unknown | - |
| IVIA 3619.1 | *Xanthomonas* sp. | Spain | *Capsicum annuum* | - |
| IVIA 3287.1 | *Xanthomonas* sp. | Spain | *Prunus persica* var*. nectarina* | - |
| IVIA 3436 | *Xanthomonas* sp. | Spain | *Prunus salicina* | - |
| CFBP 3566 | *Xanthomonas* sp. | France | *Prunus armeniaca* | - |
| DAR 64858 | *Xanthomonas* sp. | Australia | *Prunus dulcis* | - |
| IVIA 1245.80 | *Agrobacterium tumefaciens* | Spain | *Prunus persica* | - |
| IVIA 1682.4b | *A. tumefaciens* | Spain | *Prunus persica* | - |
| IVIA 2304.10 | *A. tumefaciens* | Spain | *Prunus persica* | - |
| IVIA 2974.3a | *A. tumefaciens* | Spain | *Prunus salicina* | - |
| IVIA 3201.2 | *A. tumefaciens* | Spain | *Prunus salicina* | - |
| IVIA 2261.2 | *Pantoea agglomerans* | Spain | *Olea europea* | - |
| IVIA 2677.2a | *Pseudomonas fluorescens* | Spain | *Lactuca sativa* | - |
| IVIA 3249.9 | *P. fluorescens* | Spain | *Lactuca sativa* | - |
| IVIA 3518.2 | *P. fluorescens* | Spain | *Petunia* sp. | - |
| IVIA 2627.3a | *P. syringae* | Spain | *Pyrus communis* | - |
| IVIA 2716 | *P. syringae* | Spain | *Prunus persica* | - |
| IVIA 3405.1-1 | *P. syringae* | Spain | *Citrus reticulata* | - |
| IVIA 3514.2 | *P. syringae* | Spain | *Citrus sinensis* | - |
| IVIA 3918.7 | *P. syringae* pv*. actinidae* | Spain | *Actinidia deliciosa* | - |

^a^ Information related to some of these strains can be found in the next references: Palacio-Bielsa et al. [9], Fischer-Le Saux et al. [33] and Bühlmann et al. [34].

^b^ CECT: Colección Española de Cultivos Tipo, Universidad de Valencia, Paterna, Valencia, España; CFBP: Collection Française de Bactéries Phytopathogènes, INRA, Angers, France; DAR: Australian Collection of Plant Pathogenic Bacteria, Rydalmere, Australia; ISF: Culture Collection of C.R.A.: Centro de Ricerca per la Frutticoltura, Roma, Italia; IVIA: Instituto Valenciano de Investigaciones Agrarias, Moncada, Valencia, España; RIPF: Research Institute of Pomology and Floriculture, Skierniewice, Poland.

^c^ LFIA results after after 10 min: + indicates positive detection; - indicates negative detection
